# Associations of a Plant-centered Diet and Lung Function Decline across Early to Mid-Adulthood: The CARDIA Lung Study

**DOI:** 10.21203/rs.3.rs-2845326/v1

**Published:** 2023-04-26

**Authors:** Robert Wharton, Jing Gennie Wang, Yuni Choi, Elliot Eisenberg, Mariah K. Jackson, Corrine Hanson, Bian Liu, George R. Washko, Ravi Kalhan, David R. Jacobs, Sonali Bose

**Affiliations:** Icahn School of Medicine at Mount Sinai; The Ohio State University Wexner Medical Center; University of Minnesota Twin Cities; Mount Sinai Hospital; University of Nebraska Medical Center; University of Nebraska Medical Center; Icahn School of Medicine at Mount Sinai; Brigham and Women’s Hospital; Northwestern University Feinberg School of Medicine; University of Minnesota Twin Cities; Mount Sinai Hospital

**Keywords:** diet, longitudinal changes in lung function, lung function in epidemiology, epidemiological study, lung function, respiratory epidemiology

## Abstract

**Background:**

Lung function throughout adulthood predicts morbidity and mortality even among adults without chronic respiratory disease. Diet quality may represent a modifiable risk factor for lung function impairment later in life. We investigated associations between nutritionally-rich plant-centered diet and lung function decline across early and middle adulthood from the Coronary Artery Risk Development in Young Adults (CARDIA) Study.

**Methods:**

Diet was assessed at baseline and years 7 and 20 of follow-up using the validated CARDIA diet history questionnaire. Plant-centered diet quality was scored using the validated A Priori Diet Quality Score (APDQS), which weights food groups to measure adherence to a nutritionally-rich plant-centered diet 1 to 5 points for 20 beneficially rated foods and 5 to 1 points for 13 adversely rated foods. Scores were cumulatively averaged over follow-up and categorized into quintiles. The primary outcome was lung function decline, including forced expiratory volume in 1 second (FEV_1_) and functional vital capacity (FVC), measured at years 0, 2, 5, 10, 20, and 30. We estimated the association of APDQS with annual pulmonary function changes in a repeated measures regression model, adjusting for clinically relevant covariates.

**Results:**

The study included 3,787 Black and White men and women aged 18–30 in 1985–86 and followed for 30 years. In multivariable repeated measures regression models, individuals in the lowest APDQS quintile (poorest diet) had declines in FEV_1_ that were 1.6 ml/year greater than individuals in the highest quintile (35.0 vs. 33.4 ml/year, ß±SE per 1 SD change APDQS 0.94 ± 0.36, p = 0.009). Additionally, declines in FVC were 2.4 ml/year greater in the lowest APDQS quintile than those in the highest quintile (37.0 vs 34.6 ml/year, ß±SE per 1 SD change APDQS 1.71 ± 0.46, p < 0.001). The association was not different between never and ever smokers (p_int_ = 0.07 for FVC and 0.32 for FEV_1_). In sensitivity analyses where current asthma diagnosis and cardiorespiratory fitness were further adjusted, results remained similar.

**Conclusions:**

In this 30-year longitudinal cohort study, long-term adherence to a nutritionally-rich plant-centered diet was associated with slower decline in lung function, highlighting diet quality as a potential treatable trait supporting long-term lung health.

## Background

Lung function is an important predictor of morbidity and mortality even among adults without chronic respiratory disease(1, 2). Across the general population, lifetime lung function trajectories have been categorized as persistently poor, worsening, preserved impaired, preserved good, or preserved ideal lung health(3). As lung function trajectory over the lifespan is a major determinant of the development of future chronic lung disease(3), the early identification of modifiable risk factors is critical.

Emerging evidence suggests that high quality plant-centered diets are associated with improved respiratory health. For example, a diet high in fruits and vegetables has been demonstrated to be associated with improved lung function among individuals without respiratory disease(4). In another study of smokers without respiratory disease, greater adherence to a Western diet pattern, with higher consumption of red and cured meats and sweets, and lower consumption of fruits, vegetables, legumes, and fish, was associated with increased risk of impaired lung function(5). Diets high in fruits and vegetables were associated cross-sectionally with a lower prevalence of current wheeze in children(6) and higher FEV_1_(4) in adults. In a meta-analysis of mostly cross-sectional studies of fruit and vegetable intake on prevalent wheeze and asthma severity, fruit intake was negatively associated with prevalent wheeze and asthma severity, and vegetable intake was negatively associated with prevalent asthma(7). While there is an established relationship between long-term consumption of a nutritionally-rich plant-centered diet and cardiovascular mortality throughout adulthood(8), its longitudinal association with lung health has not been explored. To address this gap, we tested the hypothesis that the consumption of a nutritionally-rich plant-centered dietary pattern is associated with improved lung function trajectory across early and middle adulthood among participants from The Coronary Artery Risk Development in Young Adults (CARDIA) Study.

## Methods

### Study design, setting and participants

CARDIA is a prospective multi-center cohort study of 5,115 young adults from four United States cities: Birmingham, Alabama; Chicago, Illinois; Minneapolis, Minnesota, and Oakland, California. Participants were 18–30 years old at baseline and were followed for 30 years with 71% retention at year 30. There were no exclusion criteria. Participants were randomly selected and recruited by telephone from census tracts in Minneapolis and Chicago, by telephone exchanges within the Birmingham city limit, and from lists of the Kaiser-Permanente Health Plan membership in Oakland and Berkeley(9). The study protocol has been published elsewhere (10).

### Assessment Of Plant-centered Diet Quality

Diet was assessed at years 0, 7, and 20 using the validated interviewer-administered CARDIA diet history. Adherence to a nutritionally-rich plant-centered diet was captured using the validated A Priori Diet Quality Score (APDQS), which weights 46 food groups rated *a priori* as beneficial, neutral, or adverse on the basis of current understanding of their known associations with cardiovascular risk. Higher scores, indicating better diet quality, are driven mainly by intake of nutritionally-rich plant foods. Plant-based foods such as fruits, avocado, green and yellow vegetables, and whole grains contribute to a higher score and are scored positively, whereas negatively scored foods include refined carbohydrates, red meats, processed meats, soft drinks, and high-fat dairy products. While the main contributors to a higher score are plant foods, certain animal products, including nonfried fish and poultry, also contribute, in recognition of the nutritious value of some non-plant-based foods. Details of the APDQS have been previously described (11).

### Assessment Of Outcome Variables

The primary outcome was lung function, including forced expiratory volume in 1 second (FEV_1_), forced vital capacity (FVC), and FEV_1_/FVC ratio. Lung function was measured at years 0, 2, 5, 10, 20, and 30 using standard procedures per European Respiratory Society and American Thoracic Society guidelines(12). Extensive quality control of the measurement devices was carried out during each exam as well as between examinations, using waveform analysis to check comparability when a different device was used in one exam than in another(13). Lung function decline was calculated by subtracting spirometry parameters at year 30 from those parameters at each participant’s estimated peak lung function and dividing by the difference in years, as has been done previously in this cohort(14). If year 30 data were not available, year 20 data were used. Obstructive lung physiology, defined as a ratio of FEV_1_ to FVC < 0.7, was a secondary outcome assessed only in smokers.

### Other Covariates

Demographics and clinical data included age (years), sex, maximal educational attainment (highest grade completed), race (Black, White), cigarette smoking and pack year history, height, weight, field center, and total daily energy intake. Smoking status was assessed yearly. Previous studies of CARDIA participants have shown strong correlation between self-reported cigarette smoking and year 0 cotinine measurements(15). Cardiorespiratory fitness (assessed as treadmill time in seconds at years 0, 7, and 20) and history of asthma were included in sensitivity analyses.

### Statistical analysis

Baseline descriptive statistics were reported according to quintiles of APDQS. We used a mixed linear model (SAS PROC MIXED) to evaluate associations between APDQS averaged over follow-up and pulmonary function changes, including FEV_1_, FVC, and FEV_1_/FVC. This averaging approach, where dietary data is remeasured or carried forward and serially averaged up to and including each measurement of spirometry, allows for minimization of random within-person error, better reflects the cumulative, long-term effect of diet, and preserves sample size. Annual pulmonary function changes, including FEV_1_, FVC and FEV_1_/FVC, were estimated per one standard deviation change in APDQS (SD = 13 points) in repeated measures regression models, adjusting for sex, education, race, peak lung function, smoking status (measured at every annual follow-up), pack-year history (years 0, 2, 5, 7, 10, 15, and 20), height, BMI, total energy intake (averaged over the time period prior to spirometry), and site. We adjusted models for age squared as prior work has shown lung function to have a quadratic decline(16). As a sensitivity analysis, we additionally adjusted for current asthma and cardiorespiratory fitness, which we conceptualized as confounders with influence on both diet and lung function. Cardiorespiratory fitness has been previously shown to be associated with lung function trajectory(17). If data on exposures and outcomes of interest were missing, participants were excluded. For continuous covariates (height and cardiorespiratory fitness), mean values were assigned if data were missing. To account for patients who had missing spirometry because of death before year 30, we evaluated the slope of FEV_1_% predicted among participants who died before year 30 by adding the interaction of death status with time to the main model.

Given that smokers may have important differences in both dietary pattern and susceptibility to environmental influences on lung function, we also tested for an interaction between smoking status and diet.

For the outcome of airflow obstruction, Cox proportional-hazards regression models were created for incident obstructive lung physiology according to quintiles of the APDQS. Hazard ratios were adjusted for the same covariates.

Lifetime trajectories of percent predicted lung function were generated using a group-based trajectory modeling approach (SAS PROC TRAJ), previously described by Washko et al.(3), which fits a mixture model via maximum likelihood. Participants were assigned *a priori* to one of the five trajectories derived from the model as persistently poor, worsening, preserved impaired, preserved good, or preserved ideal lung health(3), then stratified by APDQS quintiles.

We further investigated the relationships of APDQS with lung function measurements at each exam cross-sectionally using a linear mixed effect approach with repeated APDQS assessments and repeated covariate measurements. The models were fitted to the repeated measures of lung function, with random intercepts and fixed slopes of APDQS×time interaction using the same covariates as the primary model. All covariates were time-varying except for race, sex, and height.

For the outcome of airflow obstruction, Cox proportional-hazards regression models were created for incident obstructive lung physiology according to quintiles of the APDQS. Hazard ratios were adjusted for the same covariates. All analyses were conducted using SAS version 9.4 (SAS Institute Inc., Cary, NC).

## Results

### Study Population

A total of 3,787 participants were included in this study, as shown in Table 1. We excluded participants who withdrew consent (n = 1), lacked outcome measures (n = 1,243), or lacked exposure measures at Y0 (n = 132), leaving 3787 participants as a final sample. Ten participants (0.3%) and 24 (0.6%) were missing data on height and cardiorespiratory fitness, respectively. At enrollment, compared with the participants in the lowest APDQS quintile, those in the highest quintile were older, were more likely to be female and White, had higher maximal educational attainment, lower BMI, and lower energy intake, were less likely to be a current smoker, and had higher baseline FEV_1_ and FVC. Among all participants, 2627 (69.4%) had dietary information at all three timepoints, 967 (25.5%) had 2 measurements, and 193 (5.1%) had 1 measurement. Dietary intake strongly tracked over time. For example, Year 0 APDQS had a correlation about 0.63 and 0.58 with Year 7 and Year 20 APDQS, respectively. The correlation between Year 7 and Year 20 was 0.64.

### Lung Function Trajectory Groups

**Figure 1:**
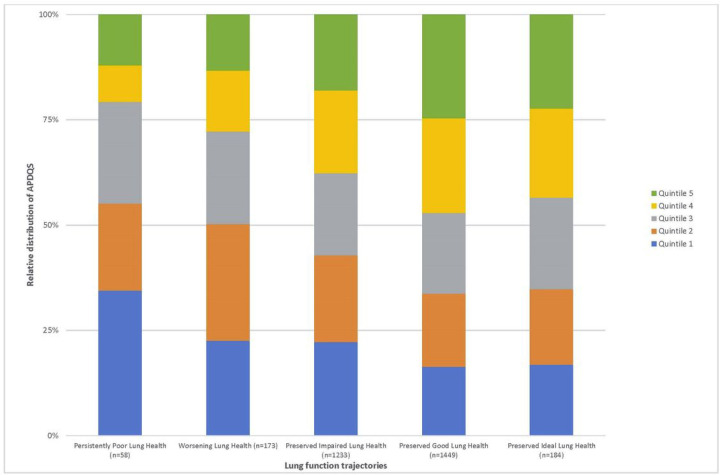
Relative distribution of APDQS quintiles among different lung function trajectories (FEV_1_ % predicted). Only participants with year 30 data and at least one other timepoint (n=3097) were included to ensure that trajectories reflected lung function changes into middle age. Quintile 5 APDQS was more represented in participants with preserved ideal and preserved good lung health, whereas participants with persistently poor lung health were more likely to have scores in quintile 1. The median APDQS scores were 52, 59.7, 66, 72.5, and 82 for quintiles 1, 2, 3, 4, and 5 respectively.

The groups of participants with preserved good and preserved ideal lung health had a greater proportion of highest quintile APDQS (21% and 22%, respectively) than the group with persistently poor lung health (12%), whereas the group with persistently poor lung health had the highest proportion (34%) of lowest quintile APDQS (Fig. 1).

### APDQS and Lung Function Decline

**Figure 2:**
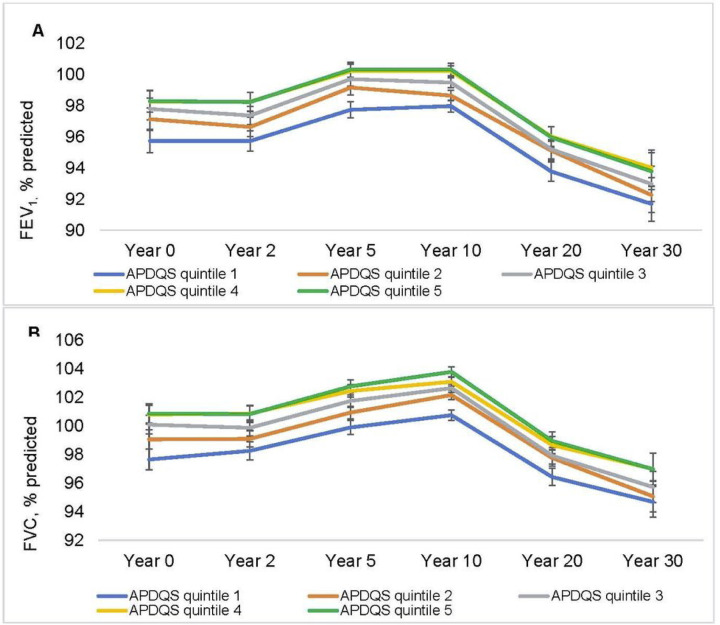
Mean pulmonary function measures (A, FEV_1_ % predicted, B, FVC % predicted) according to quintiles of the APDQS at each CARDIA exam year. Linear mixed effect models fitted to the repeated measures of lung function, with random intercepts and fixed slopes of APDQS×time interaction. N=3787 (no. of observations= 20134). Covariates included current age, time variables (Years 0, 2, 5, 10, 20, and 30), and time interactions with race (Black and White), sex, center (Birmingham, Chicago, Minneapolis, and Oakland), maximal educational attainment, height, total energy intake (Years 0, 7, 20), BMI (Years 0, 2, 5, 7, 10, 15, and 20), and lifetime pack-years of smoking (Years 0, 2, 5, 7, 10, 15, and 20). All covariates were time-varying except for race, sex, and height. 1 SD was 13.

In multivariable repeated measures regression models, there were significant associations between APDQS and annual changes in both FVC and FEV_1_ (Table 2). Individuals in the lowest (poorest diet quality) APDQS quintile had declines in FEV_1_ that were 1.6 ml/year greater than individuals in the highest (best diet quality) quintile (35.0 vs. 33.4 ml/year; ß±SE per 1 SD change APDQS, 0.94 ± 0.36, p = 0.009) and declines in FVC that were 2.4 ml/year greater than those in the highest quintile (37.0 vs 34.6 ml/year; ß±SE per 1 SD change APDQS, 1.71 ± 0.46, p < 0.001). APDQS was not significantly associated with FEV_1_/FVC. The association was not different between never and ever smokers (p_int_ = 0.07 for FVC and 0.32 for FEV_1_). In sensitivity analyses where current asthma diagnosis and cardiorespiratory fitness were further adjusted, results remained similar. At cross-sectional analyses at each time point across young and middle adulthood, higher APDQS was associated with higher FEV_1_ and FVC (Fig. 2 and Table 3).

As expected, those with fewer FEV_1_ measures had a higher death rate. In addition, those who died during follow-up had a faster decline in lung function than those who survived. Nevertheless, in a sensitivity analysis, accounting for those who died before year 30 and their trend in FEV_1_ or FVC predicted did not substantially alter the associations between APDQS and change in FEV_1_ or FVC (ß±SE 0.91 ± 0.36 (p = 0.01) and 1.67 ± 0.46 (p < 0.001), respectively).

Lastly, there was no significant difference in the development of incident obstructive lung physiology across quintiles of APDQS in any of the models (Table 4).

## Discussion

In this 30-year follow-up longitudinal study, we found that a nutritionally-rich plant-centered diet was associated with significantly less decline in lung function, even after adjustment for demographic and lifestyle factors influencing lung health. We found a difference of 1.6 ml/year decline in FEV_1_ when comparing participants in the 1st and 5th quintiles of APDQS. Putting this into context, one cohort study estimated the excess FEV_1_ decline from every 10 pack years of smoking at 2.5 ml/yr(18), while current asthma has been associated with excess FEV_1_ decline of 3.7–9 ml/year(18–20). Importantly, smoking status did not significantly modify the benefits of consuming a nutritionally-rich plant-centered diet.

Consistent with these findings, a higher proportion of high quality APDQS was observed in participants with preserved good and preserved ideal lung health trajectories than in patients with worsening or persistently poor lung health. Consequently, consumption of a typical American diet that is nutrient poor and rich in processed, calorie-dense animal products may substantially contribute to the population burden of excess lung function decline and associated morbidity and mortality throughout adulthood. It is worth noting the possibility that higher lifetime lung function observed among those in the top quintiles of plant-centered diet intake may in part be a function of reaching and/or sustaining optimal peak lung health after adolescence, and hence may reflect nutritional exposures occurring in the prenatal or childhood life stages and altering respiratory programming(21). Research on critical windows is necessary to develop dietary recommendations for children and young adults to prevent adverse long-term respiratory outcomes.

Mechanistically, plant-centered diets rich in fruits and vegetables contain antioxidants (vitamin C, flavonoids, and carotenoids), which attenuate oxidative stress and may play a role in the pathogenesis of COPD(22). In addition, dietary fiber, a key component of plant-based foods, has been shown to attenuate inflammatory responses(23), possibly through alterations in the gut microbiome and increased production of anti-inflammatory metabolites such as short-chain fatty acids(24). In a study of mice exposed to cigarette smoke, a high fiber diet decreased interleukin-6 and interferon-gamma in bronchoalveolar lavage and serum samples, attenuated development of emphysema, and was protective against alveolar destruction(25). An analysis of plant-based flavonoids and age-related decline in lung function from the Veteran’s Administration Normative Aging Study found that anthocyanins, a subclass of flavonoids found primarily in berries, were strongly associated with less age-related decline in FEV_1_ and FVC for the highest quartile of intake compared with the lowest(26)‥ On a micronutrient level, a nine-year longitudinal study of participants in Nottingham, England found that higher intake of vitamin C – abundant in fruits and vegetables – was associated with a lower rate of FEV_1_ decline by 50.8 mL per 100 mg of vitamin C(27). Thus, individual components of a nutritionally-rich plant-centered diet, including vitamins, minerals, fibers, and phytochemicals may work synergistically to provide beneficial effects on lung function (28).

### Strengths and limitations

Our study has several important strengths. Follow-up was over an extended period, with excellent retention and repeated spirometry over 30 years, as well as repeated diet information collected through an interviewer-administered diet history. By capturing adults in early to middle adulthood, we gained insight into early influences on lung function decline. The APDQS provides pragmatic, achievable pathways to healthy eating, reinforcing fruit and vegetable intake without excluding animal products. Smoking status was assessed annually with previous evaluations demonstrating a high degree of correlation between self-report of smoking status and cotinine concentrations(15).

There are a few limitations of the study worth noting. Residual confounding is impossible to eliminate, though we carefully adjusted for relevant covariates, including accounting for both the time-varying presence and extent of smoking behaviors. Diet questionnaires, while administered by trained interviewers, were ultimately self-reported and subject to recall bias. The CARDIA cohort comprised only White and Black participants, limiting generalizability to other races/ethnicities. The small number of years with complete diet data limits assessment of change in diet over time, which might influence outcomes. The study had 71% retention at year 30, and participants with missing data may have been prognostically different. Participants for whom year 20 lung function data were used may not have had time to develop significant lung function decline, since lung function is usually maintained to around age 40; however, this would be expected to bias toward the null. Finally, the trajectory analysis is limited by its descriptive nature and smaller numbers of participants in the highest and lowest quintiles of APDQS. Further analyses could elucidate which specific foods contributed most to the primary outcome, the impact of dietary changes over the life course, and whether results differed by sex. Replication in an independent cohort would strengthen causal inferences. Since our study was not powered to determine the effect of plant-centered diet quality in smokers, future work should pay close attention to this vulnerable subgroup.

## Conclusions

Although the absolute effect size of unhealthy diet on lung function (1.6 ml/year excess FEV_1_ decline) appears modest, it is important to consider diet as a lifelong, modifiable exposure. Together with efforts targeted at conventional risk factors for lung function decline such as smoking and poorly controlled chronic lung disease, public health initiatives emphasizing the importance of a nutritionally-rich, plant-centered diet early in adulthood may add to our arsenal of strategies to preserve lung health across the life course. Larger population-level analyses to confirm and expand upon these findings are warranted.

## Figures and Tables

**Table 1:** Baseline characteristics (Year 0) of participants according to quintiles of the Year 0 APDQS

	Total participants	APDQS					
		Quintile 1 (n = 720)	Quintile 2 (n = 817)	Quintile 3 (n = 773)	Quintile 4 (n = 737)	Quintile 5 (n = 740)	P-value^a^
APDQS, mean ± SD	63.5 ± 13.1	45.9 ± 4.5	55.5 ± 2.3	62.9 ± 2	70.6 ± 2.5	83.2 ± 5.8	
Age Y0, mean ± SD, y	25.1 ± 3.6	23.2 ± 3.7	24.6 ± 3.7	25.3 ± 3.6	25.7 ± 3.2	26.5 ± 2.9	< 0.001
Female, no (%)	2150 (56.8)	378 (52.5)	432 (52.9)	426 (55.1)	404 (54.8)	510 (68.9)	< 0.001
Self-identified race, no (%)							
Black	1792 (47.3)	558 (77.5)	538 (65.9)	386 (49.9)	227 (30.8)	83 (11.2)	< 0.001
White	1995 (52.7)	162 (22.5)	279 (34.2)	387 (50.1)	510 (69.2)	657 (88.8)	
Maximal educational attainment, mean ± SD, grades ^b^	15.7 ± 2.6	14.6 ± 2.4	15 ± 2.5	15.6 ± 2.6	16.4 ± 2.5	17.1 ± 2.2	< 0.001
Study center, no (%)							
Birmingham, AL	882 (23.3)	253 (35.1)	249 (30.5)	185 (23.9)	127 (17.2)	68 (9.2)	< 0.001
Chicago, IL	836 (22.1)	163 (22.6)	178 (21.8)	158 (20.4)	168 (22.8)	169 (22.8)	
Minneapolis, MN	975 (25.8)	155 (21.5)	192 (23.5)	203 (26.3)	214 (29.0)	211 (28.5)	
Oakland, CA	1094 (28.9)	149 (20.7)	198 (24.2)	227 (29.4)	228 (30.9)	292 (39.5)	
Height, mean ± SD, cm	170.3 ± 9.5	169.9 ± 10	170.3 ± 9.9	170.4 ± 9.3	170.9 ± 9.5	169.7 ± 8.7	0.16
BMI, mean ± SD, kg/m^2^	24.5 ± 4.9	24.9 ± 5.8	24.9 ± 5.3	24.9 ± 5.1	24.2 ± 4.4	23.5 ± 3.6	< 0.001
Smoking, no (%)							
Never	2247 (59.7)	463 (64.9)	477 (58.7)	455 (59.2)	435 (59.5)	417 (56.7)	< 0.001
Former	521 (13.9)	50 (7)	79 (9.7)	99 (12.9)	117 (16.0)	176 (23.9)	
Current	994 (26.4)	201 (28.2)	256 (31.5)	215 (28)	179 (24.5)	143 (19.4)	
Pack-years smoking at Y0, mean ± SD, pack-years	2.1 ± 4.3	1.9 ± 4.5	2.3 ± 4.3	2.2 ± 4.5	2 ± 4.3	1.9 ± 3.9	0.26
Pack-years smoking through Y20, mean ± SD, pack-years	5.1 ± 9.4	5.4 ± 10	6.2 ± 10.4	5.5 ± 10.1	4.6 ± 8.4	3.5 ± 7.1	< 0.001
Total energy intake, mean ± SD, kcal	2738 ± 1269	3050 ± 1351	2841 ± 1387	2785 ± 1334	2588 ± 1153	2420 ± 968	< 0.001
Physical activity, mean ± SD, EU ^c^	415.5 ± 292.9	349.7 ± 275.5	369.6 ± 289.6	406.5 ± 286.2	433.8 ± 272.5	521.6 ± 308.9	< 0.001
Cardiorespiratory fitness, mean ± SD, treadmill time, second	591.5 ± 170.9	560.2 ± 168.5	558.1 ± 172	576.5 ± 171.1	616 ± 164.2	649.4 ± 159.7	< 0.001
History of asthma, no (%)	179 (4.7)	32 (4.5)	39 (4.8)	32 (4.2)	34 (4.6)	42 (5.7)	0.77
FEV1, median (IQR), ml	3470 (2950–4100)	3280 (2810–3950)	3350 (2850–4040)	3480 (2980–4110)	3640 (3080–4260)	3540 (3130–4170)	< 0.001
FVC, median (IQR), ml	4170 (3530–5040)	3960 (3280–4780)	4020 (3370–4880)	4190 (3520–5105)	4370 (3670–5330)	4250 (3780–5130)	< 0.001
Ratio FEV/FVC, median (IQR), ml	0.835 (0.792–0.873)	0.843 (0.797–0.885)	0.838 (0.796–0.878)	0.835 (0.793–0.872)	0.829 (0.784–0.863)	0.833 (0.788–0.865)	< 0.001
Ratio FEV1/FVC < 0.7 (Obstructive lung disease), no (%)	125 (3.5)	22 (3.3)	22 (2.9)	34 (4.6)	26 (3.6)	21 (2.9)	0.39

APDQS, A Priori Diet Quality Score; BMI, body mass index; IQR, interquartile range; FEV1, forced expiratory volume in 1 second; FVC, forced vital capacity; SD, standard deviation.

a Evaluated with chi-square tests for categorical variables and ANOVA for continuous variables.

b Cumulative data through Y30.

c Exercise units, physical activity score derived from the CARDIA physical activity history.

**Table 2:** Association between APDQS and annual changes in pulmonary function measures a

	Estimated slopes per 1 SD higher APDQS ^b^	
	*ß±SE*	P-value
**FVC, ml**		
Mean ± SD	−35.8 ± 20	
MV model ^c^	1.71 ± 0.46	< 0.001
MV model + cardiorespiratory fitness ^d^	1.32 ± 0.47	0.005
MV model + current asthma ^d^	1.72 ± 0.46	< 0.001
**FEV**_**1**_ **, ml**		
Mean ± SD	−34.4 ± 15.8	
MV model ^c^	0.94 ± 0.36	0.009
MV model + cardiorespiratory fitness ^d^	0.68 ± 0.36	0.06
MV model + current asthma ^d^	0.98 ± 0.36	0.006
**FEV**_**1**_ **/FVC ratio**		
Mean ± SD	−3.1 ± 2.5	
MV model ^c^	−0.02 ± 0.06	0.67
MV model + cardiorespiratory fitness ^d^	−0.04 ± 0.06	0.49
MV model + current asthma ^d^	−0.02 ± 0.06	0.66

a (Year 30 FVC - peak FVC)/(Year 30 - peak year). Other measures were calculated in the same way. If measurements at Year 30 were not available, Year 20 data were used.

b 1 SD = 13.

c Multivariable-adjusted linear regression model adjusted for peak pulmonary function variable (depending on outcome of interest), age squared, sex, race (Black and White), center (Birmingham, Chicago, Minneapolis, and Oakland), maximal educational attainment, baseline height, averaged total energy intake, averaged BMI, and life-time pack years of smoking.

d Time-updated covariates were used (Years 0, 2, 5, 7, 10, 15, and 20).

**Table 3:** Cross-sectional association between the APDQS and pulmonary function measures at each exam year

	Estimated slopes per 1-SD higher of the APDQS^b^	
	*ß±SE*	P-value
**FVC, % predicted**		
Year 0	1.33 ± 0.2	< 0.001
Year 2	1.16 ± 0.19	< 0.001
Year 5	1.22 ± 0.18	< 0.001
Year 10	1.38 ± 0.21	< 0.001
Year 20	1.18 ± 0.23	< 0.001
Year 30	1.34 ± 0.24	< 0.001
**FEV1, % predicted**		
Year 0	1.06 ± 0.21	< 0.001
Year 2	1.15 ± 0.21	< 0.001
Year 5	1.14 ± 0.2	< 0.001
Year 10	1.32 ± 0.23	< 0.001
Year 20	1.11 ± 0.25	< 0.001
Year 30	1.26 ± 0.26	< 0.001
**FEV1/FVC ratio, %**		
Year 0	−0.39 ± 0.13	0.003
Year 2	−0.11 ± 0.13	0.37
Year 5	−0.2 ± 0.12	0.10
Year 10	−0.14 ± 0.14	0.32
Year 20	−0.18 ± 0.15	0.23
Year 30	−0.22 ± 0.16	0.18

a Linear mixed effect models fitted to the repeated measures of lung function, with random intercepts and fixed slopes of HEI-2015×time interaction. N = 3787 (no. of observations = 20134). Covariates included current age, time variables (Years 0, 2, 5, 10, 20, and 30), and time interactions with race (Black and White), sex, center (Birmingham, Chicago, Minneapolis, and Oakland), maximal educational attainment, height, total energy intake (Years 0, 7, 20), BMI (Years 0, 2, 5, 7, 10, 15, and 20), and lifetime pack-years of smoking (Years 0, 2, 5, 7, 10, 15, and 20). All covariates were time-varying except for race, sex, and height.

b 1 SD was 13.

**Table 4.** Multivariable-adjusted HRs (95% CIs) of incident obstructive lung disease according to quintiles of the APDQS among ever smokers, N = 1645

	APDQS						
	Quintile 1	Quintile 2	Quintile 3	Quintile 4	Quintile 5	Per 1 SD higher APDQS^a^	P for trend^b^
APDQS median	53	60.3	66.6	73.7	83		
Unadjusted cumulative incidence % (n/N)^c^	20.4 (66/323)	17.7 (59/334)	12.9 (42/326)	15.9 (54/339)	18.3 (59/323)		
Unadjusted HR	1 (ref)	0.96 (0.67 − 1.36)	0.63 (0.42 − 0.94)	0.89 (0.62 − 1.28)	0.93 (0.65 − 1.33)	0.96 (0.84 − 1.09)	0.54
MV model HR^d^	1 (ref)	0.96 (0.67 − 1.38)	0.74 (0.48 − 1.12)	1.08 (0.71 − 1.62)	1.13 (0.73 − 1.77)	1.04 (0.88 − 1.23)	0.67
MV model HR + cardiorespiratory fitness ^e^	1 (ref)	0.95 (0.66 − 1.36)	0.70 (0.45 − 1.07)	1.06 (0.70 – 1.61)	1.18 (0.74 − 1.86)	1.05 (0.88 − 1.25)	0.60
MV model HR + current asthma ^e^	1 (ref)	0.95 (0.66 − 1.36)	0.71 (0.47 − 1.08)	1.05 (0.70 − 1.59)	1.11 (0.71 − 1.73)	1.03 (0.87 − 1.22)	0.71

a 1 SD was 13.

b Derived by testing a continuous APDQS variable in the model.

c Cumulative incidence of obstructive lung disease from Year 2 through Year 30

d Cox proportional-hazards regression model adjusted for age, sex, race (Black and White), center (Birmingham, Chicago, Minneapolis, and Oakland), maximal educational attainment, baseline height, time-updated averaged total energy intake (Years 0, 7, 20), time-updated averaged BMI (Years 0, 2, 5, 7, 10, 15, and 20), and time-updated life-time pack years of smoking (Years 0, 2, 5, 7, 10, 15, and 20).

e Time-updated covariates were used (Years 0, 2, 5, 7, 10, 15, and 20).

## Abbreviations

CARDIA: Coronary Artery Risk Development in Young Adults
APDQS: A Priori Diet Quality Score
FEV_1_: Forced expiratory volume in 1 second
FVC: Functional vital capacity
COPD: chronic obstructive pulmonary disease
